# Polyphenol-rich açaí seed extract exhibits reno-protective and anti-fibrotic activities in renal tubular cells and mice with kidney failure

**DOI:** 10.1038/s41598-022-24420-1

**Published:** 2022-12-02

**Authors:** Elisa Bernardes Monteiro, Natalia Alvarenga Borges, Mariana Monteiro, Ângela de Castro Resende, Julio Beltrame Daleprane, Christophe Olivier Soulage

**Affiliations:** 1 Nutrition and Genomics Laboratory, Basic and Experimental Nutrition Department, Institute of Nutrition, Rio de Janeiro State University, Rio de Janeiro, 20550-900 Brazil; 2 Laboratório de Alimentos Funcionais, Nutrition Institute, Federal University of Rio de Janeiro, Rio de Janeiro, 21941590 Brazil; 3 Laboratory of Cardiovascular Pharmacology and Medicinal Plants, Department of Pharmacology, Rio de Janeiro State University, Rio de Janeiro, 20551030 Brazil; 4 CarMeN, UMR INSERM U.1060, INRAe U1397, INSA-Lyon, Université Claude Bernard Lyon 1, Groupement Hospitalier Est, Bâtiment B13, 59 Boulevard Pinel, 69500 Bron, France

**Keywords:** Kidney, Mouse, Nephrology, Chronic kidney disease, Renal fibrosis

## Abstract

The main goal of this study was to evaluate the reno-protective effects of a phenolic-rich Açaí seed extract (ASE) in mice with kidney failure. Kidney failure was induced chemically with an adenine-rich diet (0.25% w/w for 4 weeks) in male CD1 Swiss mice. Mice were then provided daily with ASE (at a dose of ~ 350 mg/kg/day) in drinking water for 4 weeks. Adenine mice exhibited renal dysfunction evidenced by increased proteinuria, increased uremia, extensive tubular atrophy and kidney fibrosis associated with overexpression of pro-fibrotic genes (collagen 1a1, transforming growth factor β1, TGF-β1) and markers of tubular injury (such as Kidney injury molecule-1, KIM-1). ASE was able to beneficially counteract all these effects. ASE improved oxidative damage and fibrosis by decreasing carbonylated protein and MDA concentrations, as well as collagen deposition in renal tissue. ASE decreased the expression of TGF-β1 gene and the abundance of protein TGF-β1 in kidneys. It further decreased both expression and urinary excretion of tubular injury biomarkers, e.g., KIM-1 and Neutrophil gelatinase-associated lipocalin. CKD ASE-treated mice exhibited higher polyphenol content and total antioxidant capacity compared to control mice. ASE further prevented the expression of profibrotic genes in HK2 human tubular cells exposed to uremic toxins. Taken together, these findings suggest that ASE exerted potent reno-protective and anti-fibrotic effects through its antioxidant activity and the modulation of the TGF-β1 pathway.

## Introduction

Chronic kidney disease (CKD) is characterized by the occurrence of kidney damage along with a gradual and irreversible loss of kidney function. It often coexists with cardiovascular diseases (CVD), diabetes and hypertension, and is an important public health issue in most countries around the world^1^. Kidney function decline is translated into diminished glomerular filtration rate, proteinuria, and increased serum creatinine levels^2^. Among the factors that could possibly contribute to progressive renal failure, oxidative stress and inflammation play a crucial role. Oxidative damage in kidney can be induced by reactive oxygen species (ROS), which activate the nuclear factor-kappa B (NF-κB) pathway; as a result, both oxidative stress and pro-inflammatory cytokine production occur, thus leading to systemic inflammation and tubulointerstitial fibrosis^3–5^. In this context, bioactive compounds have emerged in recent years as useful dietary tools that are able to prevent oxidative damage and inflammation in CKD^6–8^.

Several studies have shown the beneficial effects of polyphenols in accomplishing a reno-protective dietary approach^9–13^. Açaí (*Euterpe oleracea* Mart.) is a tropical berry from the Amazon region in Brazil that has been gained extensive attention for its antioxidant and anti-inflammatory actions in different experimental models of chronic diseases. An extract from açaí seed, rich in phenolic compounds (açaí seed extract or ASE), was described to exert antihypertensive effects and to prevent endothelial dysfunction and vascular structural disorders in two-kidney-one-clip (2K1C) renovascular hypertension^14^. ASE was also shown to prevent insulin resistance and hepatic steatosis in a murine model of obesity^15,16^. Additionally, ASE has proven to attenuate endothelial dysfunction and diminish oxidative and inflammatory burden in endothelial cells by beneficial modulation of antioxidant defense enzymes, pro-inflammatory cytokine expression and activation of nuclear factor erythroid 2-related factor 2 (Nrf2) pathway, one of the most important transcription factors involved in antioxidant and cytoprotective responses^17,18^.

In this scenario, the present study aims to explore the reno-protective effects of ASE in a murine model of kidney failure as well as in an in vitro model of uremia using human renal tubular cells.

## Material and methods

### Chemicals and reagents

Unless mentioned, all chemicals were purchased from Sigma-Aldrich (Saint-Quentin Fallavier, France). All organic solvents were purchased from Carlo-Erba Reagents (Peypin, France) and were of HPLC grade.

### Açaí seed extract (ASE)

Fruits from the Amazon region (Belém do Pará, Brazil, 2° 57′ 29, 47″ S/47° 23′ 10, 37″ W) were processed to obtain the hydro-alcoholic extract (thereafter referred to as ASE) as previously described^19^. Açaí (*Euterpe oleracea* Mart.) is registered in IPNI Life Sciences Identifier (LSID) number 666941-1, collation ii. 29. tt. 29, 30.

The phenolic compound profile of ASE was previously reported by our group and is shown in Supplementary Table 1^16,17^.

### Animals and experimental design

All experimental procedures were performed in accordance with the guidelines laid down by the French Ministry of Agriculture (no. 2013-118) and the European Union Council Directive for the protection of animals used for scientific purposes of September 22nd, 2010 (2010/63UE). The study protocol was approved by the local ethic committee (CETIL, Comité Ethique de l’INSA-Lyon, CNREEA no 102) on February 29th, 2016 under the reference *Apafis # 3210-2015121608242729v1*. The present study was reported in accordance with the *ARRIVE 2.0 Essential 10* guidelines (https://arriveguidelines.org). Forty male CD1 Swiss mice (21 days of age) were purchased from Janvier-Labs S.A. (Le Genest-Saint-Isle, France) and housed in an air-conditioned room with a controlled environment of 21 ± 0.5 °C and 60–70% humidity, under a 12-h light/dark cycle and with free access to food and water.

**Figure 1:**
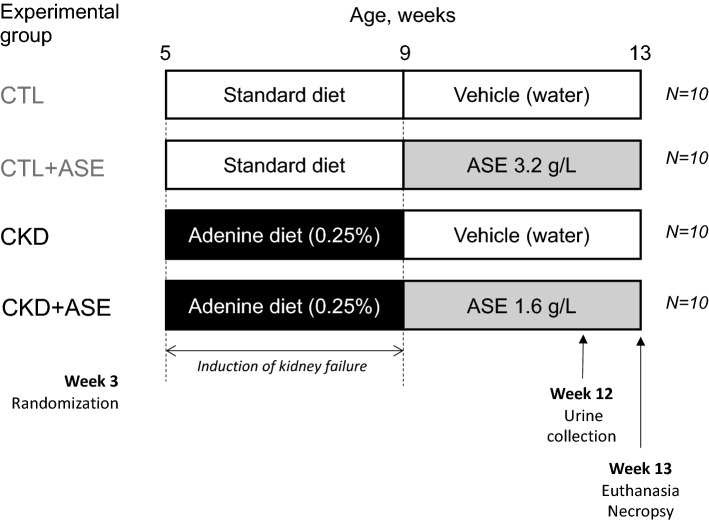
Schematic representation of the experimental design.

Mice were allowed to acclimatize for 2 weeks before induction of renal failure. Renal failure was induced chemically by feeding the animals for four weeks with an adenine-containing diet (0.25% w/w; SAFE custom diet, Augy, France) as described by^20^. Animals were randomly divided into 4 groups of 10 mice using the R software package *blockrand*(https://www.rdocumentation.org/packages/blockrand/versions/1.5/topics/ blockrand), namely: (1) Control group, which received a commercial standard diet and drinking water throughout the whole experiment (n = 10 mice); (2) Control group supplemented with ASE (Control + ASE), which received a standard diet throughout the experiment and ASE-containing drinking water (3.2 g ASE/L of water) for the last 4 weeks of the experiment (n = 10 mice); (3) CKD group, which received an adenine-containing diet for 4 weeks, followed by a standard diet in the subsequent 4 weeks (n = 10 mice); (4) CKD group treated with ASE (CKD + ASE), which received an adenine-containing diet for 4 weeks, followed by a standard diet and ASE-containing drinking water (1.6 g ASE/L of water) for the last 4 weeks of the experiment (n = 10 mice). Figure 1 shows the experimental design of the study. The standard commercial diet used in this study was composed of 46.7% carbohydrates, 3.9% fibers, 16.1% proteins, 3.1% lipids (w/w) and exhibited energetic density of 14.0 kJ/g (A04 diet, Safe Diets, Augy, France). The chosen dose of ASE has already been shown to reduce oxidative stress and inflammation in mice, as previously described by our group^15,21^. Note that since the uremic mice exhibited a twofold higher water intake than control mice (see Fig. 3), the concentration of ASE was halved (i.e., 1.6 vs 3.2 g/L) to ensure a similar daily intake of ASE in both groups. Body weight were monitored on a weekly basis. Water intake was measured twice weekly to calculate the actual daily ASE intake and dose.

### Urine collection

Every two animals from each group were housed into metabolic cages (Charles River laboratory, L’Arbresle, France) for collection of 24-h urine. 24 h-diuresis and water intake were measured gravimetrically. Urine was centrifuged for 10 min at 1500 × *g*, and the supernatant was collected and frozen at − 20 °C.

### Animal euthanasia and necropsy

At the end of eight weeks, animals were anesthetized with sodium pentobarbital (400 mg/kg) and euthanized by cervical dislocation for subsequent removal of organs and tissues. Body weight (BW) and length were measured. Tissues and organs were dissected out, weighted, snap frozen in liquid nitrogen, and stored at − 80 °C until analysis. Blood was collected by cardiac puncture in a heparinized syringe and centrifuged (3500 × *g*, 2 min) for plasma separation. Plasma was immediately frozen in liquid nitrogen and stored at − 80 °C until analysis. One kidney was fixed by immersion for 48 h in 4% (w/v) paraformaldehyde in buffered phosphate for histological studies.

### Renal function parameters and biochemical analyses

Blood glucose (in the fed and fasted state) was determined by collecting a drop of blood from the terminal portion of the tail and using an Accu-Check® Performa Glucometer (Roche, Meylan, France). Renal function was evaluated by the following parameters: daily water intake, 24 h-diuresis, 24 h-proteinuria and plasma concentration of urea. Urine protein concentration was determined with the Bradford assay^22^ using bovine serum albumin (BSA) as a standard. Plasma concentrations of urea, total cholesterol and triglycerides were quantified using commercial kits from Sobioda (Montbonnot-Saint-Martin, France) and bioMérieux (Marcy l’Etoile, France). Total phenolic content in plasma was assayed according to the method of Folin–Ciocalteu as described by Swain and Hillis^23^ using gallic acid as a standard. Total antioxidant activity of plasma was measured by the Ferric Reducing Ability of Plasma (FRAP) assay as described by Benzie and Strain^24^.

### Oxidative damage biomarkers

Quantification of carbonylated proteins was determined in hepatic and renal tissues based on the formation of carbonyl group by reaction with 2,4-dinitrophenylhydrazine (DNPH), performed according to^25^. Malondialdehyde (MDA) was quantified in renal and hepatic tissues in accordance to Grotto et al.^26^. Briefly, malondialdehyde content was determined using high performance liquid chromatography (HPLC) coupled with diode array detector and spectrum in the UV–visible detection. In both experiments, results were normalized to total protein content estimated by Bradford assay^22^.

### Renal histology

Kidneys were embedded in paraffin, cut and stained with Sirius red. Pictures were taken with an Olympus microscope and analyzed using the ImageJ software (https://imagej.nih.gov/ij/) to quantify fibrosis. The mean glomerular area was quantitated using the open-source Qpath software (https://qupath.github.io/)^27^. The tubular atrophy score was defined as follows: 0 = normal tubules, 1 = rare single atrophic tubule, 2 = several clusters of atrophic tubules; and 3 = massive tubular atrophy. All histological measurements were performed by investigators blinded to the experimental group.

### Analysis of gene expression

Kidney tissues were crushed into liquid nitrogen, and RNA was extracted using the TRIzol Reagent (Sigma-Aldrich). Purity and concentration of RNA were determined using a Tecan reader and a NanoQuant plate (Tecan, Lyon, France). First-strand cDNAs were synthesized from 1 µg of total RNAs using a PrimeScript RT kit (Ozyme, Saint-Cyr-l’École, France). Real-time PCR assays were performed with Rotor-Gene 6000 (Qiagen, Courtaboeuf, France) using SYBR qPCR Premix Ex Taq (Ozyme). The TATA-box binding protein (TBP) was used as reference gene to normalize the results. Primer sequences are listed in Supplementary Table 2.

### Immunoassays

Kidney tissues were crushed into liquid nitrogen and resuspended in PBS. Mouse TGF-β1 was assayed using a beta 1 EIA Kit (reference ab119557, Abcam, France) according to the manufacturer’s recommendations. Urine concentrations of kidney injury molecule-1 (KIM-1), and neutrophil gelatinase-associated lipocalin (NGAL) were assayed with commercially available EIA kits from R&D systems (Abingdon, UK). 8-Hydroxydeoxyguanosine (8-OH-dG) was assayed using commercially available EIA kit from Clinisciences (Nanterre, France).

### Collagen deposition in renal tissue

Collagen content in renal tissue was determined by using the Hydroxyproline Assay kit (ref MAK-008, Sigma-Aldrich) using a spectrophotometer at 560 nm wavelength (MultiskanGo, Thermo Fisher Scientific, MA, USA), and hydroxyproline concentration was determined using a *cis*-4-hydroxy-L-proline standard curve. Collagen content was calculated assuming that 13.5% of the collagen molecule is composed by hydroxyproline^28^.

### Characterization of phenolic metabolites in plasma and urine

Phenolic compounds metabolites profile in plasma and urine were determined in the two ASE treated groups. Plasma phenolic metabolite extraction was performed as follows: sample aliquots (200 µL) were added to HCl 12 N (20 µL), distilled water (1 mL) and ethyl acetate (6 mL). Afterwards, samples were homogenized for 10 s and centrifuged at 1300 × *g* for 10 min. The supernatant was then removed (6 mL) and ethyl acetate was added (3 mL) for a second extraction. After this procedure, the supernatant was removed (3 mL) and evaporated under N_2_ flux at 38 °C, and the dry residue was kept frozen at − 20 °C until analysis.

In urine samples, solid phase extraction (SPE) was performed using Waters SPE Oasis HLB 3 mL. The cartridge was activated with 2 mL of methanol and washed with 2 mL of distilled water. After this procedure, the samples were diluted (1:1, *v/v*) in acidified aqueous solution (H_3_PO_4_ 4%) and loaded into the cartridge. In Control + ASE group, a clarification step was performed twice by centrifuging the samples at 1300 × *g* for 10 min, prior to cartridge loading. Washing steps were carried out with 2 mL of water/methanol solution (95:5, *v/v*) and elution was performed with 2 mL of methanol containing formic acid (1 mL/L). After extraction, the samples were collected, evaporated in liquid nitrogen at 38 °C, and the dry residue was kept frozen at − 20 °C until analysis.

Urine and plasma samples were resuspended, respectively, in 500 µL and 350 µL of eluent A (0.3% formic acid and 1% acetonitrile in water). Phenolic compound standards (gallic acid, catechin, epicatechin, isovanillic acid, vanillic acid, 4-hydroxyphenylacetic acid, 3,4-dihydroxyphenylacetic acid, hippuric acid (HA), quercetin, and quercetin-3-glycoside) were purchased from Sigma-Aldrich.

The liquid chromatography system (Shimadzu®, Kyoto, Japan) included two parallel pumps LC-20AD, automatic injector SIL-20AHT, diode array detector SPD-M20A, system controller CBM-20A and degasser DGU-20A5. Chromatographic separation of phenolic compounds was achieved using a reverse phase column (C18, 5 µm, 150 mm × 2.1 mm, Kromasil®). The mobile phase consisted of a gradient of 0.3% and 1% acetonitrile in water (eluent A) and 1% acetonitrile in methanol (eluent B), at a flow rate of 1 mL/min (Supplementary Table 3). Phenolic compounds were monitored from 190 to 370 nm. Identification of all phenolic compounds was performed by comparison with retention time and absorption spectrum of the respective standard. Quantification was performed by external calibration, and results were expressed in µg of phenolic compound per mL of plasma or urine. Data were acquired by LabSolutions software (Shimadzu Corporation®, version 5.82, 2015).

### Cell experiments: HK2 human tubular cells

#### Cell culture

Human kidney-2 cells (HK-2; ATCC®CRL-2190™, Manassas, USA) were cultivated in Dulbecco’s Modified Eagle Medium (DMEM™; Gibco®, LifeTechnologies™, USA) supplemented with 1% (v/v) antibiotics streptomycin (100 mg/mL) and penicillin (100 UI/mL) (Cultilab, São Paulo, Brazil), and 10% (v/v) heat-inactivated bovine fetal serum (Cultilab, São Paulo, Brazil) at 37 °C and 5% CO_2_ humidified atmosphere. The experiments were conducted in accordance with the guidelines from the European Uremic Toxin Work Group (EUTox)^29^. Cells were treated with uremic toxins Indoxyl sulfate (IS; 63 µg/mL or 250 µM) and p-Cresyl sulfate (p-CS; 40 µg/mL or 212 µM) in concentrations corresponding to those found in humans with end-stage renal disease^30^. Since both toxins are synthesized as potassium salt, a solution of 35 µg/mL (200 µM) K_2_SO_4_ in saline was used as a control to equal the potassium concentration in the K-salt of IS and p-CS. Because both toxins are mostly protein-bound in biological systems, cell medium was supplemented with 35 g/L BSA according to the recommendations of EUTox^29^. ASE concentration was set at 10 μg/mL since it was previously described as a non-cytotoxic concentration^18^.

#### Quantitative real time PCR

Cells were cultured in 6-well plates (10^5^ cells/well) and treated with ASE (10 μg/mL), p-CS (40 μg/mL) or IS (63 μg/mL), followed by a 24 h incubation period. The cells were then washed with ice-cold PBS and RNA extraction was performed with Trizol® (1 mL/well). Total RNA was quantified using a BioDropμLITE spectrophotometer (BioDrop, UK). In a further step, RNA was converted to cDNA (complementary DNA) and quantified by real time qPCR (Applied Biosystems High-Capacity RNA-to-cDNA Kit, Life Technologies, MA, USA). Reaction parameters were considered as follows: 60 min at 37 °C; 5 min at 95 °C; cooling at 4 °C (thermocycler Veriti 96 Well Thermal Cycler, Applied Biosystems, MA, USA). TaqMan® gene expression assay (Applied Biosystems, MA, USA) for Homo sapiens (Hs) was used to detect the following parameters: Collagen 1α (Col-1α; Hs01103890_m1), transforming growth factor β1 (TGF-β1; Hs00608187_m1), connective tissue growth factor (CTGF; Hs00170014_m1), α-smooth muscle actin (α-SMA; Hs01566408_m1), and glyceraldehyde-3-Phosphate Dehydrogenase (GAPDH; Hs01548420_m1) mRNA expression. Quantitative real time PCR was performed using 7500 Fast Real-Time PCR System (Applied Biosystems, MA, USA). Primers sequences are listed in Supplementary Table 2.

### Statistical analysis

Data are expressed as mean ± 1 standard deviation (SD). Normality was evaluated using QQ plots and Shapiro–Wilk tests. Data were analyzed by two-way analysis of variance (renal status, treatment), followed when appropriated by Tukey’s tests for multiple comparisons. A decision was made to analyze the smallest datasets (n ≤ 5) with non-parametric statistics. Thus, an analysis of gene expression from cell experiments was performed using Kruskall & Wallis test, followed by Dunn’s test while phenolic compounds in plasma and urine were compared using Mann & Whitney U tests. Data were analyzed using software GraphPad Prism® version 6.0 (La Jolla, CA, USA) and the R software (https://www.r-project.org/). Differences were considered significant at the *P* < 0.05 level.

### Ethics approval

All experimental procedures were performed in accordance with the guidelines laid down by the French Ministry of Agriculture (no 2013-118) and the European Union Council Directive for the protection of animals used for scientific purposes of September 22nd, 2010 (2010/63UE). The study protocol was approved by the local ethics committee (CETIL, Comité Ethique de l’INSA-Lyon, CNREEA no 102) on February 29th, 2016 under the reference *Apafis # 3210-2015121608242729v1*.

## Results

### Biometric data

**Figure 2:**
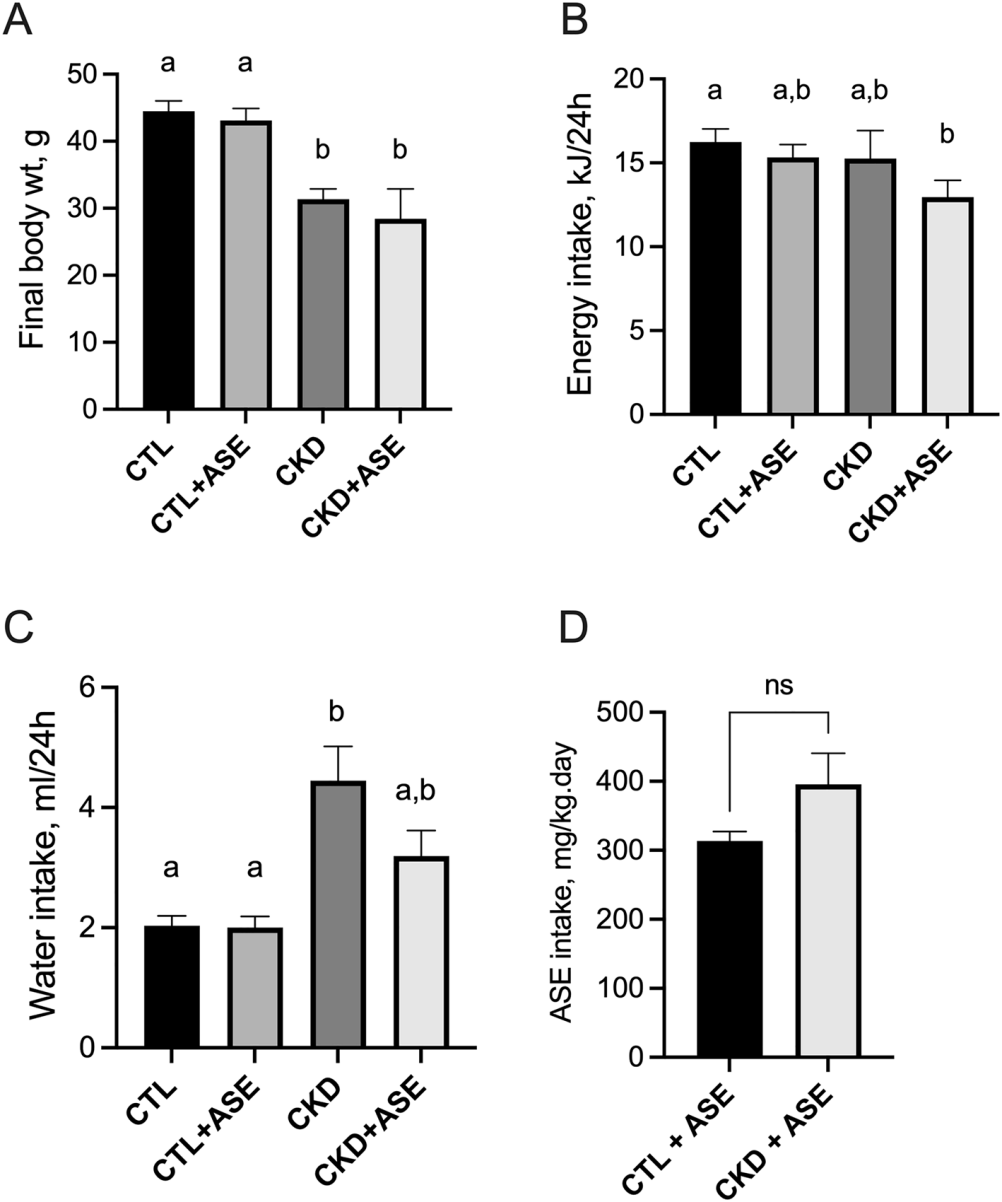
Body weight gain, cumulative water intake, and mean food intake in experimental mice. Final body weight (**A**), mean energy intake (**B**), mean water intake (**C**) and mean daily ASE consumption (**D**) in experimental mice. Data are expressed as means ± SD (n = 9–10 for each group). Significant differences were determined by two-way ANOVA followed by Tukey’s multiple comparison test. Different letters indicate a significant difference between groups at the *P* < 0.05 level.

**Figure 3:**
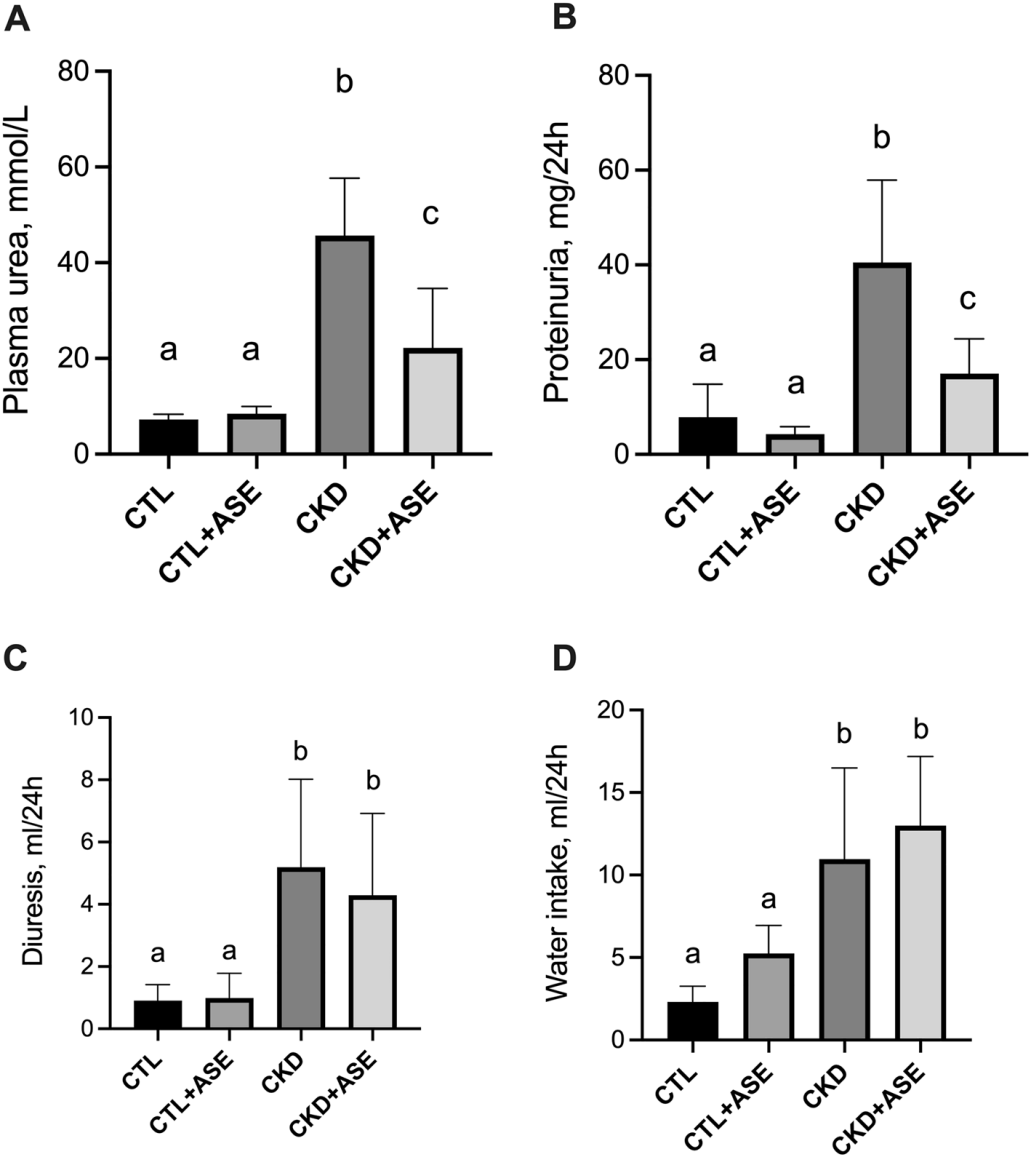
Evaluation of renal function in experimental mice. Plasma concentration of urea (**A**), daily proteinuria (**B**), 24 h-diuresis (**C**) and daily water intake (**D**). Data are expressed as means ± SD (n = 9–10 for each group). Significant differences were determined by two-way ANOVA followed by Tukey’s multiple comparison test. Different letters indicate a significant difference between groups at the *P* < 0.05 level.

Animals from both CKD groups exhibited lower body weights compared to Control groups (− 26% in CKD group; − 27% in CKD + ASE group compared to Control group, *P* < 0.001; Fig. 2A). The treatment with ASE did not influence weight gain since CKD and CKD + ASE groups showed similar weights, as found in the Control and Control + ASE groups (see growth curves in Supplementary Fig. 1). Mean daily energy intake was not different between the 4 groups (Fig. 2B, *P*  = 0.214). Both groups of CKD animals, however, exhibited a polydipsic behavior (Fig. 2C and Supplementary Fig. 2) related to their polyuria (see 24 h-diuresis in Fig. 3D). Mean daily ASE intake calculated from the daily water intake (Fig. 2D) was 314 ± 14 and 396 ± 148 mg/kg/day for control and CKD mice, respectively (*P* = 0.348). The main biometric characteristics of the animals are shown in Table 1. Animals from CKD and CKD + ASE groups exhibited a striking reduction in kidney weight when compared to Control mice (− 39% and − 36%, respectively, both *P* < 0.0001). CKD and CKD + ASE groups further showed cardiomegaly (i.e., an increase in heart weight) compared to their counterparts (+ 37% and + 39%, respectively; *P* < 0.001). Animals from both CKD groups showed a significant reduction in total body fat when compared to Control groups (reduction ranging from 45 to 65%; *P* < 0.001), as well as lower proportions of subcutaneous, epididymal and retroperitoneal white adipose tissues (Table 1). CKD mice supplemented with ASE, however, exhibited a higher adipose tissue accretion than CKD mice (+ 41%, *P* < 0.05), which suggests that these animals have a better nutritional status.

**Table 1:** Biometric data of experimental mice.

N	Control	Control + ASE	CKD	CKD + ASE	Renal st	*Treatment*	*Interaction*
	10	9	10	10			
Body weight, g	44.5 ± 1.6^a^	43.1 ± 1.8^a^	33.0 ± 1.2^b^	32.6 ± 2.0^b^	< 0.001	0.864	0.262
Length, cm	11.2 ± 0.1^a^	11.0 ± 0.1^a^	10.0 ± 0.2^b^	10.0 ± 0.2^b^	< 0.001	0.997	0.243
Liver weight, mg/10 g	482 ± 26	499 ± 17	443 ± 16	448 ± 16	0.204	0.111	0.418
Heart weight, mg/10 g	43 ± 1^a^	44 ± 1^a^	59 ± 2^b^	61 ± 2^b^	< 0.001	0.377	0.817
Kidney weight, mg/10 g	148 ± 7^a^	137 ± 7^a^	90 ± 10^b^	88 ± 4^b^	< 0.001	0.349	0.538
scWAT, mg/10 g	74 ± 8^a^	87 ± 30^a^	31 ± 6^b^	39 ± 5^b^	< 0.001	0.142	0.711
eWAT, mg/10 g	217 ± 31^a^	222 ± 96^a^	76 ± 13^b^	119 ± 16^c^	< 0.001	0.317	0.431
rWAT, mg/10 g	61 ± 8^a^	78 ± 36^a^	29 ± 5^b^	38 ± 6^b^	< 0.001	0.111	0.562
Total WAT, mg/10 g	425 ± 51^a^	475 ± 188^a^	166 ± 28^b^	235 ± 30^c^	< 0.001	0.188	0.835

Data are expressed as mean ± 1 SD. Significant differences were determined by two-way ANOVA, followed by Tukey’s multiple comparison tests. Different letters indicate a significant difference at the *P* < 0.05 level.

*ASE* Açaí seed extract, *CKD* Chronic kidney disease, *Renal st* Renal status.

### Biochemical data

The concentrations of the main plasma metabolites are shown in Table 2. Mice from both CKD groups exhibited increased glycemia in fasting as well as in fed states compared to their counterparts (*P* = 0.001). CKD + ASE mice exhibited higher fasting blood glucose in comparison to both control groups (*P* = 0.001). Plasma cholesterol concentrations were also significantly higher in both CKD groups compared to Control groups (*P* < 0.001) while there was no difference for triglycerides. No difference was found in protidemia.

**Table 2:** Biochemical data of experimental mice.

N	Control	Control + ASE	CKD	CKD + ASE	Renal st	*Treatment*	*Interaction*
	10	9	10	10			
Fasting glycemia, mmol/L	4.28 ± 0.17^a^	4.11 ± 0.22^a^	5.50 ± 0.39^a,b^	5.89 ± 0.67^b^	0.001	0.751	0.476
Fed glycemia, mmol/L	8.94 ± 0.22^a^	9.00 ± 0.28^a^	10.27 ± 0.44^b^	10.39 ± 0.50^b^	< 0.001	0.872	0.619
Triacylglycerols, mmol/L	1.43 ± 0.58	1.22 ± 0.41	0.94 ± 0.21	1.10 ± 0.50	0.900	0.138	0.364
Total cholesterol, mmol/L	2.33 ± 0.43^a^	2.26 ± 0.26^a^	2.72 ± 0.22^b^	3.17 ± 0.38^b^	0.030	0.517	0.359
Proteinemia, g/L	44 ± 3	41 ± 3	38 ± 4	38 ± 4	0.255	0.650	0.608

Data are expressed as mean ± SD. Significant differences were determined by ANOVA followed by Tukey’s multiple comparison tests. Different letters indicate a significant difference at the *P* < 0.05 level.

*ASE* Açaí seed extract, *CKD* Chronic kidney disease, *Renal st* Renal status.

### Effect of ASE on biomarkers of kidney failure

**Figure 4:**
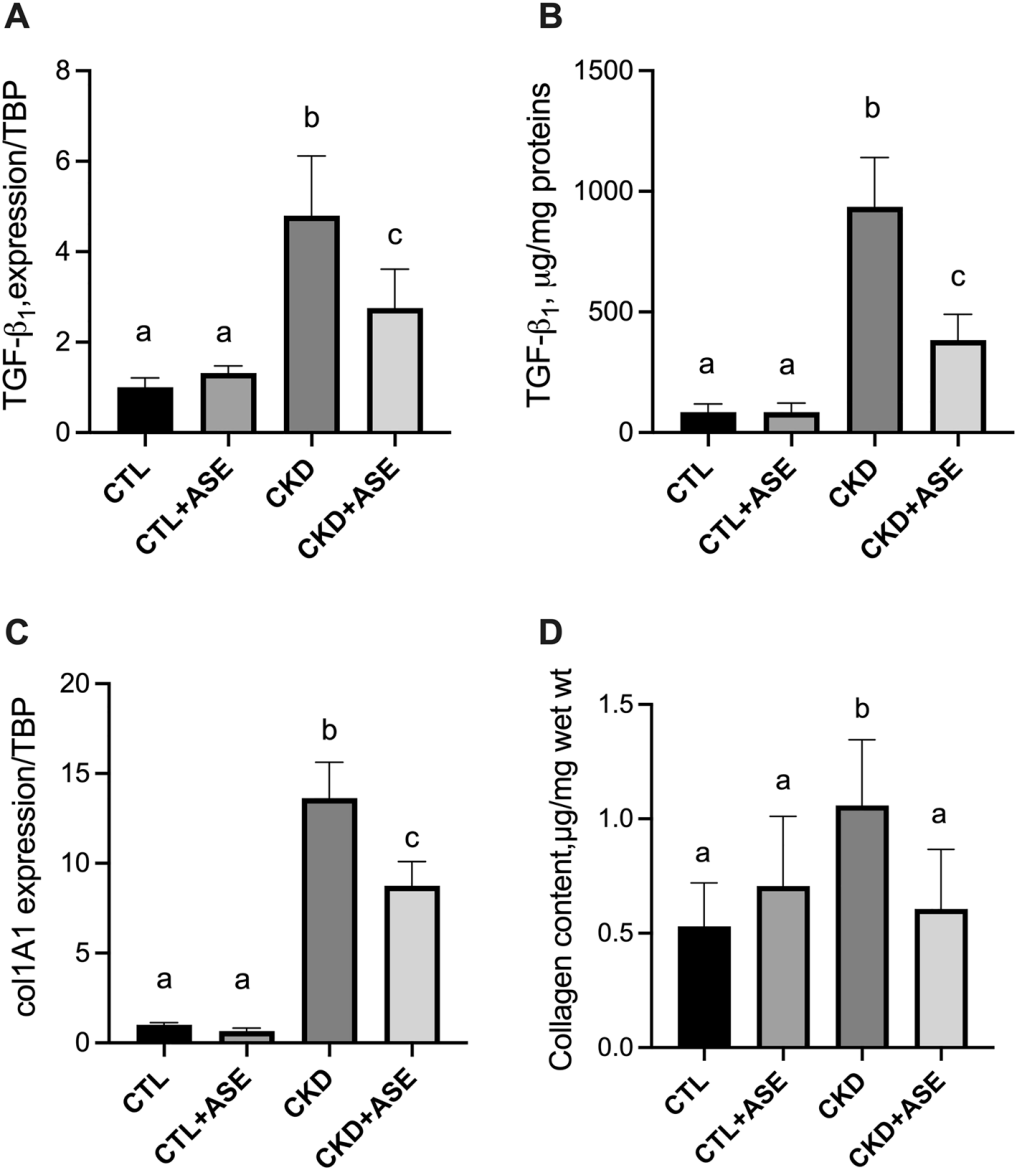
Biomarkers of kidney fibrosis. (**A**) Gene expression of transforming growth factor β1(TGF-β1), (**B**) immunoassay of TGF-β1 protein in kidney lysate. (**C**) Gene expression of collagen 1 type 1 (col1a1). (**D**) Collagen tissue content in the kidney estimated by hydroxyproline assay. TATA box binding protein (TBP) was used as reference gene to normalize all the gene expression data. Data are expressed as mean ± SD for n = 8–10 animals in each group. Significant differences were determined by two-way ANOVA followed by Tukey’s multiple comparison test. Different letters indicate a significant difference between groups at the *P* < 0.05 level.

**Figure 5:**
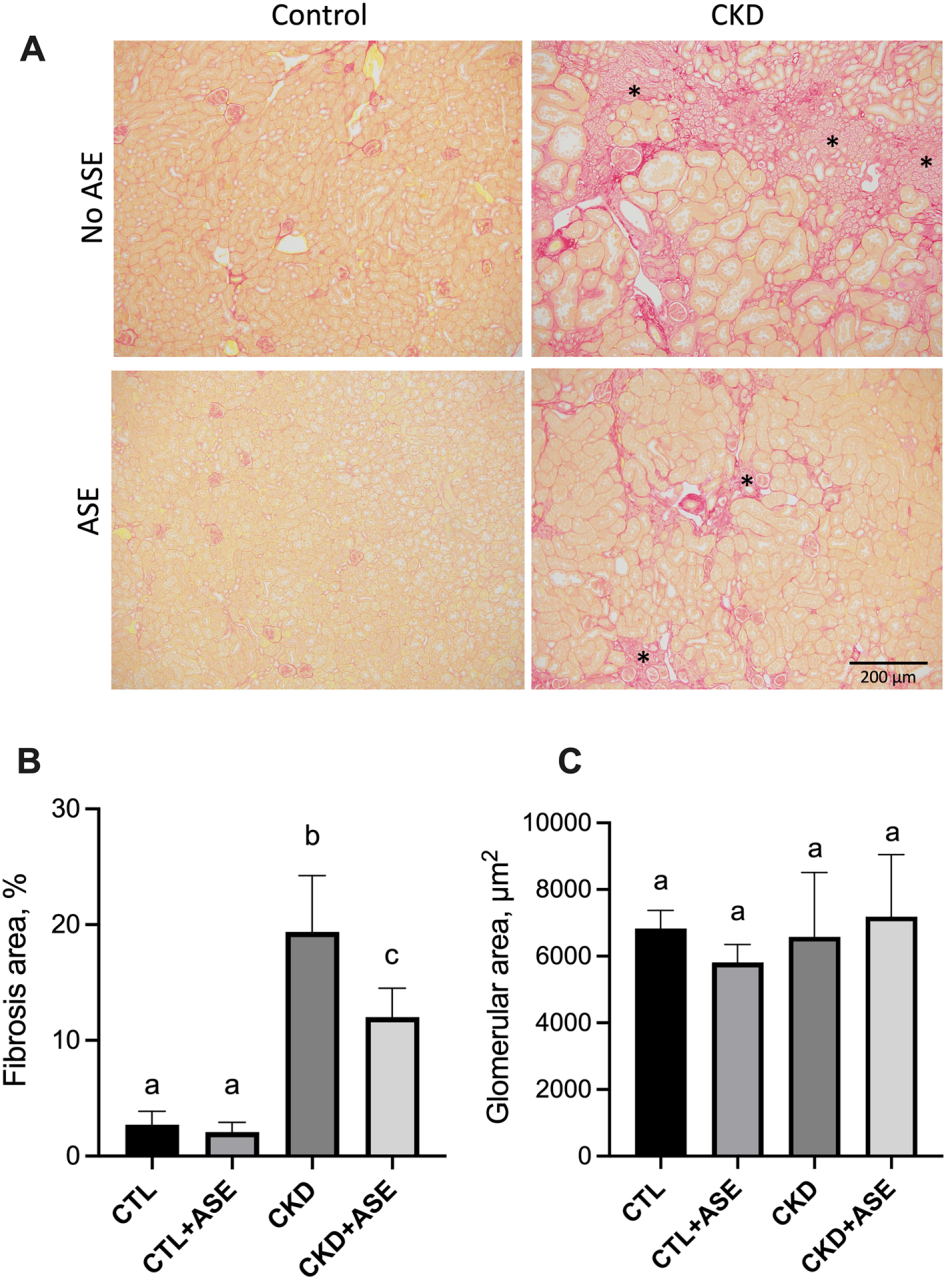
Histological evaluation of kidney fibrosis. (**A**) Typical pictures of fibrotic kidney sections in control and CKD mice. Kidney sections were stained with Sirius Red to evidence collagen deposition. Section viewed under bright field with magnification × 10. (**B**) Sirius red morphometric evaluation in control and CKD mice. (**C**) Mean surface area of glomeruli in control and CKD mice. Significant differences were determined by two-way ANOVA followed by Tukey’s multiple comparison test. Data are expressed as mean ± SD for n = 8–10 animals in each group. Different letters indicate a significant difference between groups at the *P* < 0.05 level.

**Figure 6:**
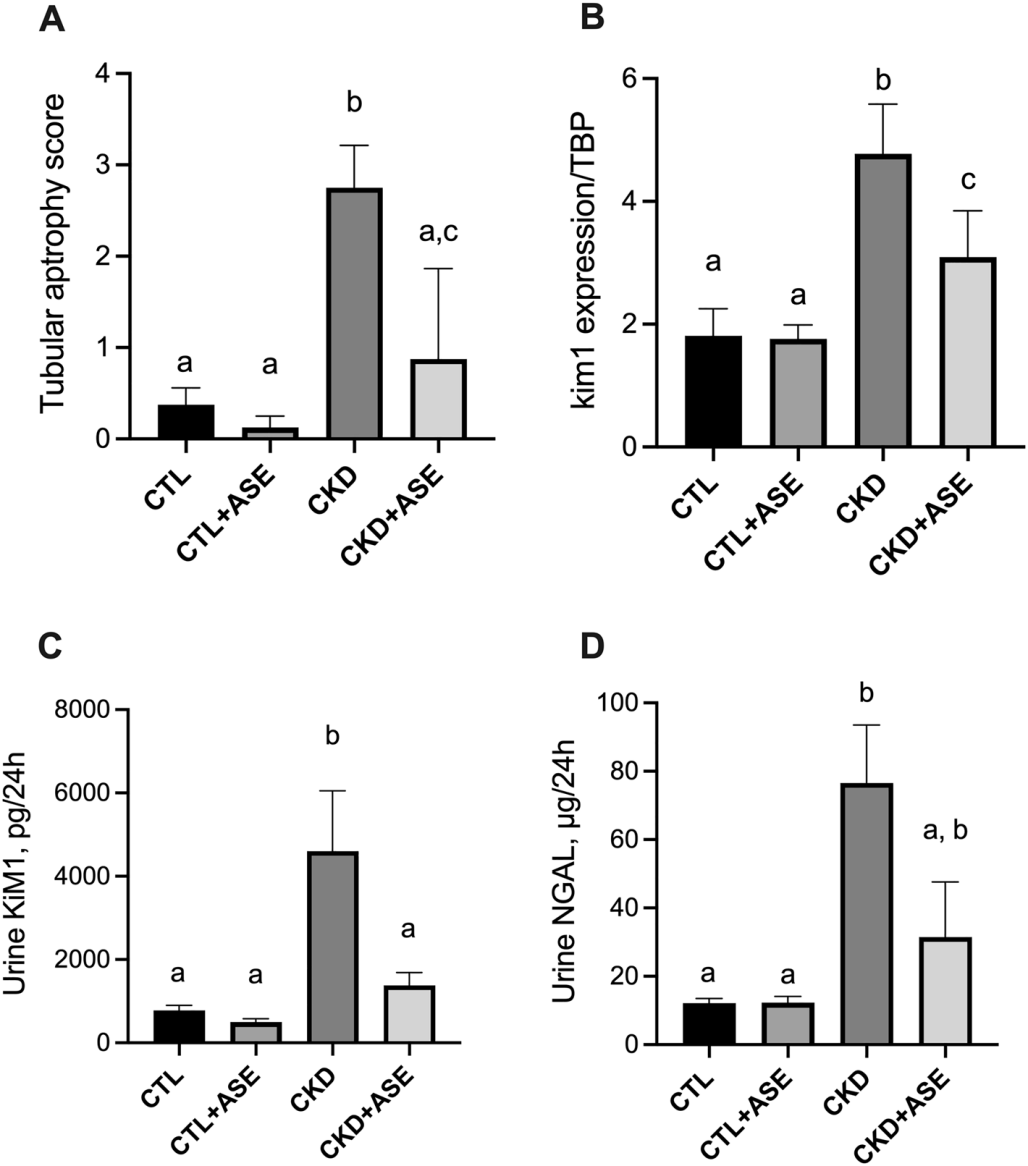
Biomarkers of tubular injury in experimental mice. Histological score of tubular atrophy (**A**) and gene expression of Kidney injury molecule 1 (Kim-1) in kidneys (**B**). Expression of Kim-1 was normalized to the expression of TATA box binding protein (TBP). Urinary excretion of two common biomarkers of tubular injury, Kim-1 (**C**) and neutrophil gelatinase-associated lipocalin (NGAL) (**D**). Significant differences were determined by two-way ANOVA followed by Tukey’s multiple comparison test. Data are expressed as mean ± SD for n = 8–10 animals in each group. Different letters indicate a significant difference between groups at the *P* < 0.05 level.

CKD animals exhibited higher level of uremia and proteinuria (two hallmarks of kidney failure) than the two control groups (Fig. 3A,B). CKD mice supplemented with ASE, however, exhibited lower plasma concentration of urea (*P* < 0.05) and lower level of proteinuria than their CKD counterparts (*P* < 0.05, Fig. 3A,B). CKD mice demonstrated polyuria (Fig. 3C) that was counter-balanced by increased water intake (Fig. 3D). Supplementation with ASE showed no effect on water intake and diuresis (*P* < 0.05; Fig. 3C,D). Importantly, 24 h water intake was strongly correlated with 24 h diuresis (r = 0.89, *P* < 0.001); thus, monitoring of water intake could be a good proxy of diuresis and change in renal function. CKD mice exhibited a strikingly increased water intake compared to control mice throughout the whole protocol (see Supplementary Fig. 2). Cumulative water intake was, however, lower in CKD mice supplemented with ASE, suggesting a protective effect on renal function. We further conducted an analysis of gene expression and protein abundance of common biomarkers of kidney fibrosis (Fig. 4). Quantitative PCR indicated a significant increase in the expression of renal fibrosis-related genes such as TGF-β1 (Transforming Growth Factor beta 1) and Col1a1 (collagen alpha-1 type 1) in the kidney of the CKD mice compared to control mice (Fig. 4A,C). In good agreement, we noticed an increased abundance of TGF-β1 protein and collagen in the kidney of the CKD mice (Fig. 4B,D) unambiguously evidencing the development of renal fibrosis after an adenine diet. In contrast, CKD mice supplemented with ASE presented a reduced level of all biomarkers associated with renal fibrosis. Kidney fibrosis was also evaluated histologically using Sirius red staining (Fig. 5A). Sirius-red positive areas were significantly increased in CKD mice compared to control animals (Fig. 5B) (2.7 ± 1.2% vs 19.4 ± 4.9%, *P* < 0.001). In contrast, CKD animals chronically fed with ASE presented a reduced area of fibrosis (12.0 ± 2.5%, *P* < 0.001 compared to CKD mice). No significant difference was found in mean glomeruli size in the four groups regardless of their renal status (Fig. 5C). There were significantly more signs of tubular atrophy (estimated by a histological injury score) in the adenine group than in the two control groups (Fig. 6A). However, CKD mice supplemented with ASE exhibited a lower tubular atrophy score than CKD mice. The expression of tubular injury biomarker KIM-1 in the kidney was significantly higher in the adenine group than in both control groups (Fig. 6B). In good agreement, urinary excretion of KIM-1 and NGAL, two common biomarkers of tubular injury, were significantly increased in the adenine group compared to the control groups (Fig. 6C,D). All these changes were significantly ameliorated by the chronic supplementation with ASE with a decrease in KIM1 expression in the kidney (− 35%, *P* < 0.05 compared to CKD) or in the urinary excretion of KIM-1 or NGAl (− 70%, *P* < 0.05 and − 59%, *P* < 0.05, respectively). Taken together, these results suggest that supplementation with ASE partially prevented kidney injury induced by the adenine diet and, in the long run, it slowed an evolution toward kidney failure.

### Effect of ASE on oxidative damage biomarkers

**Figure 7:**
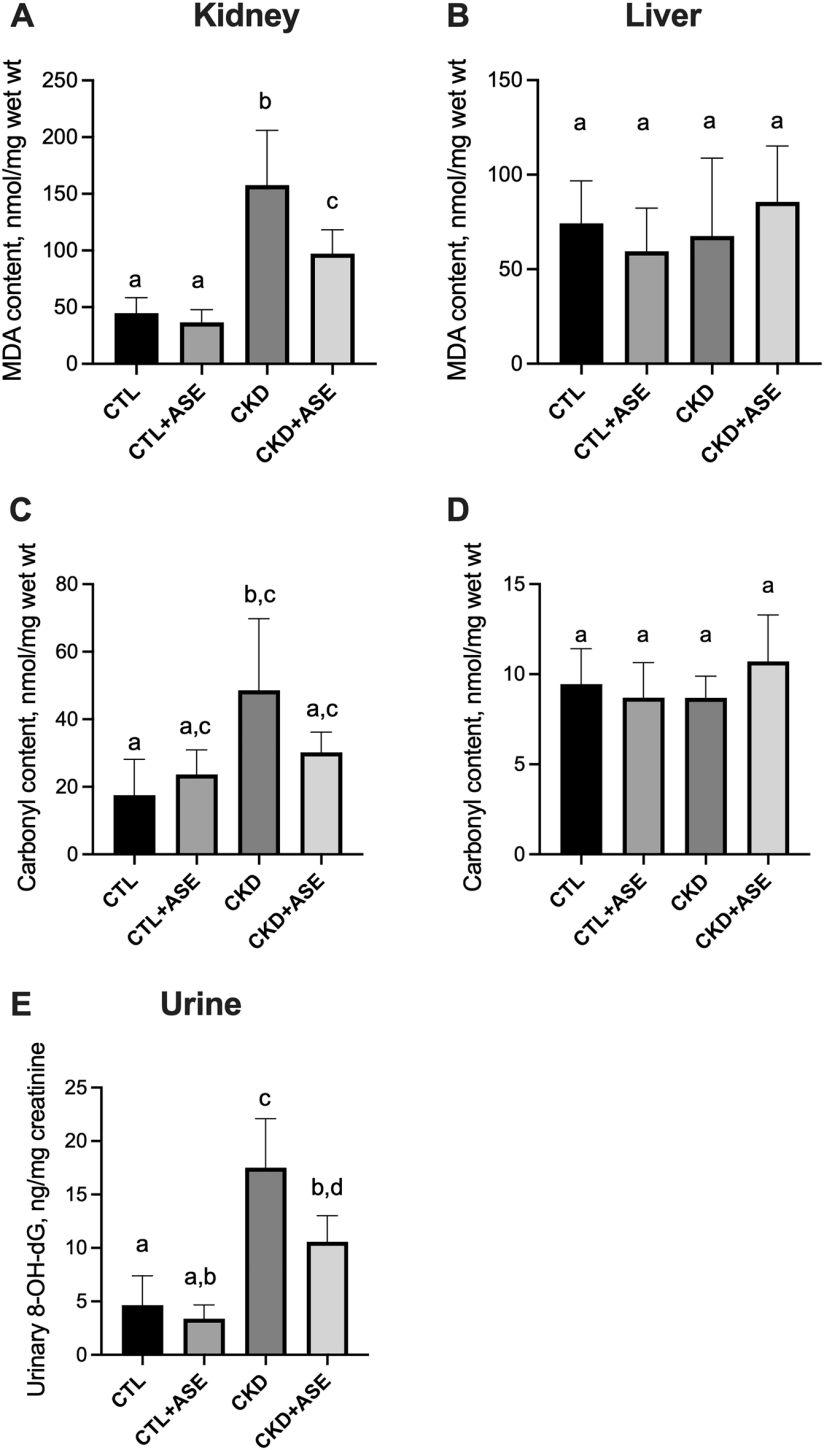
Biomarkers of oxidative damage in experimental mice. Malondialdehyde content in renal (**A**) and hepatic tissues (**B**); protein carbonyl content in renal (**C**) and hepatic tissues (**D**). Daily urinary excretion of 8-Hydroxydeoxyguanosine (8-OH-dG) (**E**). Data are expressed as means ± SD (n = 9–10 for each group). Significant differences were determined by two-way ANOVA followed by Tukey’s multiple comparison test. Different letters indicate a significant difference between groups at the *P* < 0.05 level.

Oxidative stress and inflammation are thought to play a pivotal role for adenine action and the development of kidney failure. We therefore measured two markers of oxidative insult on lipids and proteins in the kidney (the main site of adenine action) as well as on liver (taken as an organ preserved from adenine action). CKD animals exhibited higher levels of MDA and protein carbonyl than control animals in the kidney (Fig. 7A,C) (+ 250%, *P* < 0.05 from control and + 176%, *P* < 0.05 from control, respectively) but not in the liver (Fig. 7B,D) evidencing the oxidative stress associated with kidney failure. As observed in Fig. 7A,C, ASE was able to remarkably decrease MDA concentrations (− 38%, *P* < 0.05) while a trend was noticed for carbonylated protein (− 38%, not significant for CKD) in renal tissue from CKD mice; therefore, ASE prevented oxidative damage in the kidney (*P* < 0.05). In contrast, there was no difference in the liver tissues of animals supplemented with ASE (Fig. 7B,D). 8-Hydroxydeoxyguanosine (8-OH-dG) is one of the most extensively studied oxidative stress markers of DNA damage. We therefore assayed 8-OH-dG in the urine and found a striking increase of its concentration in CKD animals compared to non-CKD controls. In contrast, animals supplemented with ASE only exhibited a moderate increase of 8-OH-dG in urine (Fig. 7E).

### Phenolic compound profile in plasma and urine of experimental animals

**Figure 8:**
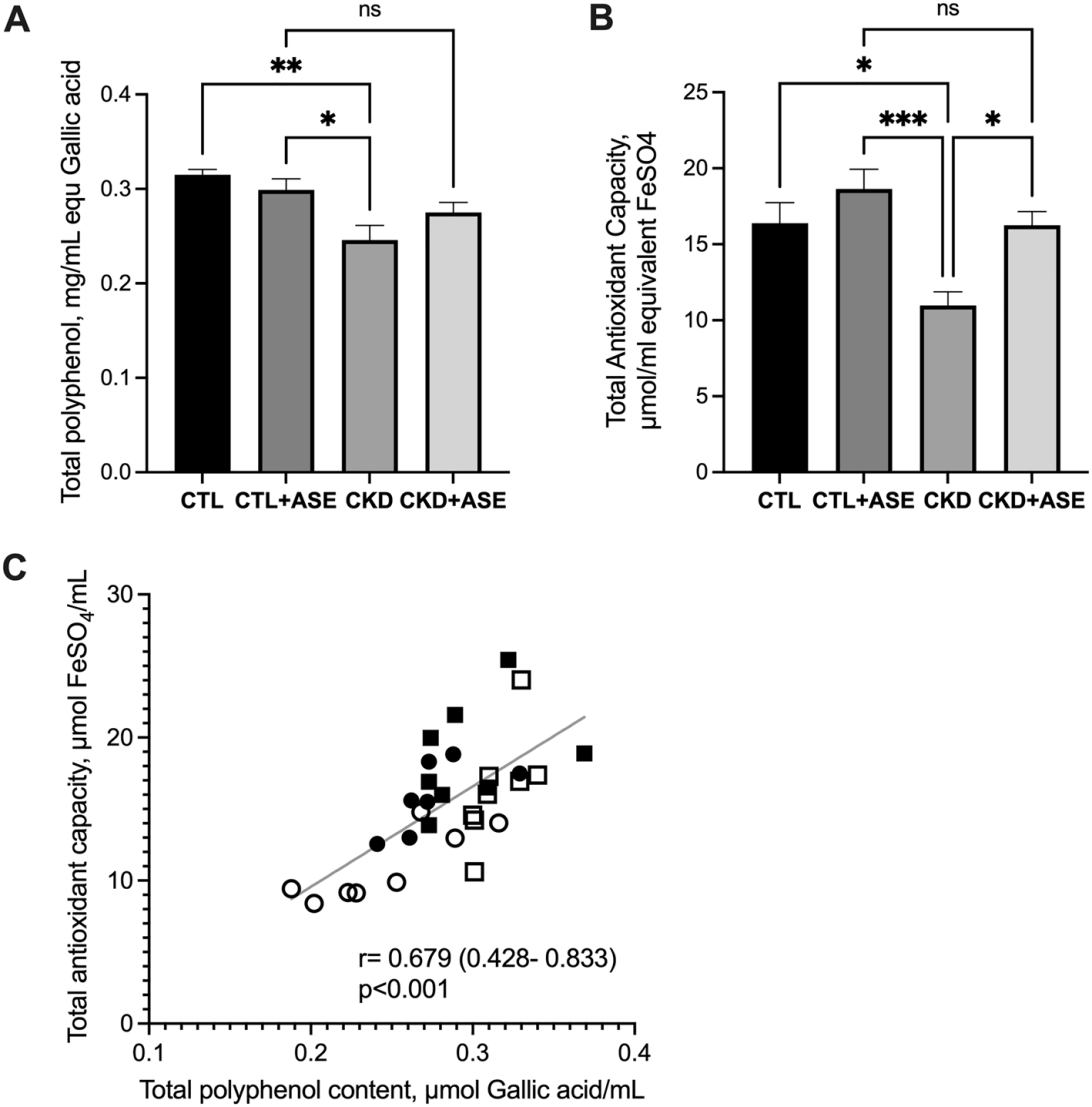
Total polyphenol content and total antioxidant capacity of plasma. (**A**) Plasma total polyphenol content was assayed according to the method of Folin–Ciocalteu using gallic acid as standard. The results were expressed as µmol equivalents of gallic acid per mL. (**B**) Plasma total antioxidant capacity evaluated by the ferric reducing ability of plasma (FRAP) assay. The results were expressed as µmol equivalents of FeSO_4_ per mL. (**C**) Relationship between plasma polyphenol content and its antioxidant capacity for control mice (open squares), control + ASE (solid squares), CKD (open circles) and CKD + ASE (solid circles) mice. Data are expressed as mean ± SD for n = 8 animals in each group. Significant differences were determined by two-way ANOVA followed by Tukey’s multiple comparison test. Different letters indicate a significant difference between groups at the *P* < 0.05 level.

To assess the effect of the supplementation with ASE, the plasma concentration of polyphenols was assayed according to the method of Folin-Ciocalteu (Fig. 8A). Total polyphenol content was lower in CKD mice than in Control mice (− 22%, *P* = 0.001). Chronic supplementation with ASE increased the plasma polyphenol content in CKD animals while there was no difference in Controls. As many polyphenols are recognized as potent antioxidants, plasma total antioxidant capacity was evaluated using the FRAP assay (Fig. 8B). CKD mice exhibited a lower plasma antioxidant capacity than Control mice (− 33%, *P* < 0.01), which was fully restored by supplementation with ASE. Interestingly, there was a good correlation between plasma concentration of polyphenols and total antioxidant capacity of plasma (Pearson’s r = 0.679, 95%CI [0.428–0.833], *P* < 0.001), suggesting that the daily intake of Açai polyphenols improved the antioxidant status of mice (Fig. 8C).

To investigate possible metabolic pathways of ASE phenolic compounds, analyses of plasma and urine metabolite were performed (Table 3). Hippuric acid was found to be the major metabolite in urine of Control + ASE and CKD + ASE mice, and its concentration was equivalent in both groups. On the other hand, 3.4-dihydroxyphenylacetic acid presented a fivefold higher concentration in CKD mice compared to its counterpart (*P* < 0.05). Catechin and vanillic acid showed higher urinary excretion in Control + ASE group (*P* < 0.05), while gallic acid presented a similar excretion rate in both groups (*P* > 0.05). Total urine metabolite concentrations were similar in both groups, although the exclusion of hippuric acid in Control + ASE samples demonstrated a superior excretion of metabolites (*P* < 0.05). Hippuric acid was detected in plasma of CKD mice in a two-fold higher concentration when compared to its counterpart (*P* < 0.05).

**Table 3:** Metabolites of phenolic compounds in plasma and urine of experimental mice.

Metabolites (µg/mL)	CTL + ASE	CKD + ASE
**Urine**		
Hippuric acid	285.6 ± 3.4	278.9 ± 3.0
3.4-dyhydroxyfenilacetic acid	4.5 ± 0.0	22.4 ± 1.0*
Catechin	35.2 ± 7.0^a^	4.7 ± 1.0*
Vanillic acid	8.8 ± 3.0^a^	nd
Gallic acid	0.02 ± 0	0.02 ± 0.0
Total	334.1 ± 29.7	306.0 ± 9.1
Total (excluding hippuric acid)	48.5 ± 6.8	27.1 ± 0.1*
**Plasma**		
Hippuric acid	7.1 ± 1.0	15.5 ± 1.0*

Data are expressed as mean ± SD (n = 4 for each group). Significant differences were determined by Mann & Whitney U tests.

*nd* Not detected, *CKD* Chronic kidney disease, *ASE* Açaí seed extract.

*Indicates a significant difference at the *P* < 0.05 level between groups.

### ASE exhibits anti-fibrotic activity on HK2 human tubular cells

**Figure 9:**
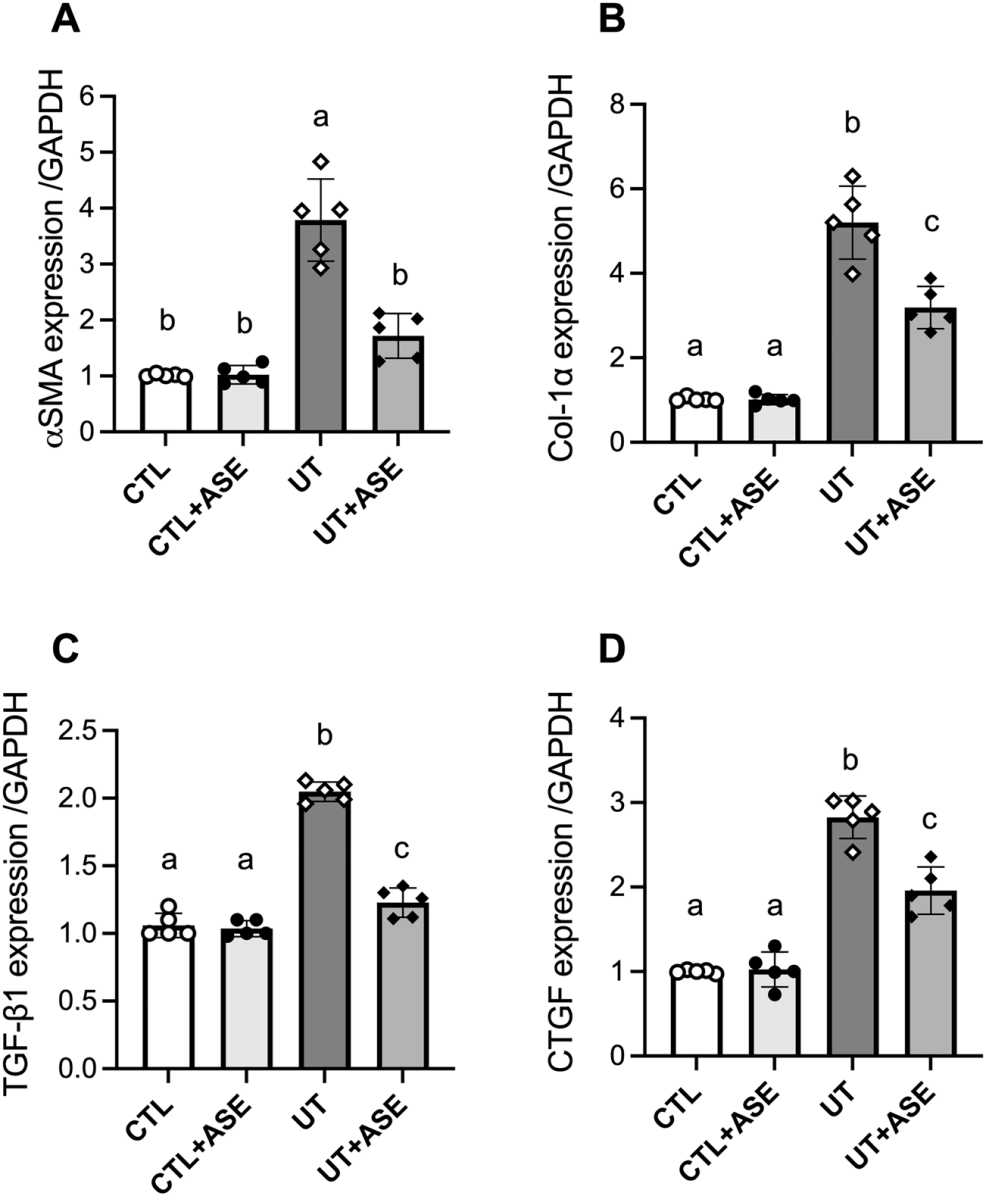
Treatment with ASE prevents the expression of profibrotic genes in human tubular cells exposed to uremic toxins. Human tubular cells HK2 were incubated for 16 h with two major uremic toxins, p-Cresyl sulfate (p-CS) and indoxyl-sulfate (IS) at concentrations found in patients with end-stage renal disease (200 and 212 µM for P-CS and IS, respectively) and expression of pro-fibrotic gene expression was evaluated. Gene expression of Collagen 1α (Col-1α) (**A**), transforming growth factor β1 (TGF-β1) (**B**), connective tissue growth factor (CTGF) (**C**) and α-smooth muscle actin (α-SMA) (**D**), All gene expressions were normalized to the expression of glyceraldehyde-3-Phosphate Dehydrogenase (Gapdh). Data are expressed as median for n = 5 independent experiments in each group. Significant differences were determined by Kruskall & Wallis test and posthoc Dunn’s tests. Different letters indicate a significant difference between groups at the *P* < 0.05 level.

We developed an in vitro model of uremia by incubating human tubular HK2 cells with two major uremic toxins at concentrations found in patients with end-stage renal disease. Also, we evaluated the effect of ASE treatment in HK-2 cells in the presence of uremic toxins Indoxyl sulfate (IS) and p-Cresyl sulfate (p-CS) on fibrotic biomarker expression (Fig. 9A–D). Incubation of HK2 cells with IS and p-CS significantly increased the expression of pro-fibrotic genes (α-SMA 3.8-fold, Col1a1 5.1-fold, TGF-β1 1.9-fold and CTGF 2.8-fold, all P-values < 0.05 compared to controls). ASE treatment significantly decreased the expression of α-SMA (− 55%, *P* < 0.05), Col-1α (− 49%, *P* < 0.05), TGF-β1 (− 38%, *P* < 0.05) and CTGF (− 30%, *P* < 0.05) of cells exposed to uremic toxins, suggesting therefore that ASE blunted uremic toxin induced pro-fibrotic gene expressions.

## Discussion

Epidemiological studies suggest that consumption of phenolic compounds can reduce the incidence of chronic diseases such as CKD, possibly owing to the bioactivity exerted by these compounds and other phytochemicals that act synergistically^31–33^. We therefore hypothesized that a polyphenol-rich extract from Açaí could be used as a potential strategy to minimize CKD burden in adenine-fed mice. Previous studies have shown that ASE exhibits many beneficial biological effects. ASE has potent antioxidant and anti-inflammatory activities in vitro as well as in vivo in experimental models of inflammation^34–36^. ASE further attenuates endothelial dysfunction and diminishes oxidative and inflammatory burden in endothelial cells^17,18^. Chronic administration of ASE improves aerobic physical performance in rats by increasing vascular and mitochondrial function^37^. ASE exerts an antihypertensive effect to prevent endothelial dysfunction and vascular remodeling in hypertensive rats^14^. ASE administration was also proved to prevent insulin resistance and hepatic steatosis in a murine model of obesity^15,16^. However, to the best of our knowledge, the effect of ASE on kidney disease has not been explored. The present study demonstrates that ASE could decrease renal damage in mice with adenine induced renal failure mainly through its anti-fibrotic and antioxidant properties.

In the current investigation, renal failure was induced in mice using an adenine diet, a model widely known to instigate tubulointerstitial damage. After intestinal absorption, adenine is metabolized to 2,8-dihydroxyadenine, which crystalizes and precipitates in renal proximal tubules. Consequently, tubular occlusion and local injury occur, which leads to inflammation of the tubular epithelium, tubulointerstitial fibrosis and renal dysfunction^38–40^. In agreement with previous reports, renal failure was successfully established in this experimental model, as evidenced by increased plasma urea concentration and proteinuria^20,41,42^. Moreover, polyuria was found in CKD mice, as evidence of reduced capacity to concentrate urines and leading to a compensatory increase in water intake. Our data showed that chronic administration of ASE decreases the high levels of plasma urea and proteinuria induced by adenine feeding. The histological tubular atrophy score showed that ASE decreased the extension of tubular damages. We further investigated the expression and urinary excretion of common biomarkers of tubular injury such as KIM-1 and NGAL. KIM-1 is recognized as a sensitive and powerful biomarker of kidney injury^43–45^. Expression of KIM-1 and urinary concentrations of both KIM-1and NGAL were increased in mice fed with adenine and decreased by chronic supplementation with ASE. CKD is often associated with tubulointerstitial fibrosis, resulting in the loss of nephrons, which are the functional units of the kidney^38^. The extension of renal fibrosis is regarded as a cornerstone in the progression of renal disease as it is a common hallmark of many renal diseases leading to renal failure. Adenine mice exhibited extensive areas of fibrosis (as evidenced histologically through Sirius red staining) and supplementation with ASE successfully mitigated this effect. Likewise, we found a remarkably higher collagen deposition in renal tissue from CKD mice compared to the control groups, as well as an increased expression of Col1a. Epithelial to mesenchymal transition (EMT) is a major step in the development of renal fibrosis^46^ and TGF- β1 was shown to play a central role in this process^47,48^. We noticed an increase of both TGF-β1 gene expression and TGF-β1 protein abundance in kidney from CKD animals. Importantly, ASE promoted a protective effect by mitigating these deleterious processes, which demonstrates a potential role in offering protection from renal fibrosis and preventing renal dysfunction, as previously described in hypertensive^49^ and diabetic rats^50^. Taken together, these observations suggest that ASE can prevent renal damage in mice fed with adenine and improve kidney function. The beneficial effects of ASE might be largely related to the inhibition of tissue fibrosis through the modulation of TGF-β1 secretion.

The progression of kidney fibrosis is intimately associated with oxidative stress. Indeed, TGF-β1 causes oxidative stress in the kidney through activation of NADPH oxidases^51–53^ that further sustains the conversion of fibroblasts to myofibroblasts (i.e. the EMT). To investigate oxidative stress in the kidney, lipid peroxidation and the measurement of carbonylation of proteins were measured. Increased MDA and carbonylated protein concentrations in renal tissue from CKD mice unambiguously evidenced the occurrence of oxidative damage; in contrast, ASE accomplished to improve this deleterious effect in CKD mice by diminishing both biomarker levels, therefore preserving cellular function. ASE further reduced the urinary excretion of 8-OH-dG, a common by-product of oxidative insult to DNA. The possible mechanisms of ASE could be related to the modulation of antioxidant enzymes (such as superoxide dismutase, catalase and glutathione peroxidase) or possibly act as a scavenger of reactive species, as reported in our previous studies^18,49,50^. The assay of plasma total polyphenol contents and total antioxidant capacity (using FRAP assay) supports this view (see Fig. 6). Indeed, renal failure in mice was associated with a decrease in plasma polyphenol content and a reduced antioxidant capacity. Both parameters were restored by daily supplementation with ASE polyphenols, and there was a good association between these two parameters (see Fig. 6C). Modulation of oxidative stress by ASE, whatever the mechanism involved, could have led to the reduction of kidney fibrosis. It is well documented that CKD in rodents promotes metabolic dysfunctions culminating in weight loss, adipose tissue depletion and ectopic lipid redistribution^54^. ASE further attenuated adipose tissue depletion in CKD (see Table 1), suggesting that it improves the nutritional status of the animals (i.e., metabolic efficiency, since food intake was similar).

Uremic toxins are deleterious compounds that accumulate in patients with renal failure as a result of decreased renal clearance^30^. Among uremic toxins, protein bound uremic toxins (PBUTs) such as IS and p-CS are the more noxious owing to their poor removal by common dialysis methods. PBUTs were described as major contributors to the progression of renal failure and potent promotor of renal fibrosis^55^. To evaluate the ability of ASE to prevent PBUT-induced fibrosis, a cellular model of uremia was implemented. Human renal tubular cells were exposed to p-CS and IS at concentrations found in patients with renal failure^30^. ASE blocked the PBUT-induced expression of pro-fibrotic genes, suggesting that part of the nephroprotective effect of ASE could result from prevention of toxicity of uremic toxins on renal tissues.

Profiling of phenolic compounds in urine and plasma of ASE supplemented animals (Control + ASE versus CKD + ASE) was carried out to evaluate the possible metabolic routes of compounds present in ASE in both physiological conditions. According to a previous study conducted by our group, ASE presents (+)-catechin and (−)-epicatechin as the main phenolic compounds, followed by (−)-epigallocatechin gallate. Such compounds belong to the subclass of flavan-3-ols, whose main metabolites are proanthocyanidins^17^. We observed that the consumption of ASE resulted in the urinary excretion of catechin and hippuric acid, vanillic acid, 3.4-dihydroxyphenylacetic acid and gallic acid. However, the excretion of these compounds occurred differently for Control and CKD mice. Mice from the Control group that consumed ASE showed greater excretion of catechin and vanillic acid compared to CKD mice that also received ASE. These compounds were possibly differentially metabolized by the intestinal microbiota of CKD mice and originate by-products that were not detected in our study. Indeed, dysbiosis (i.e., microbiota imbalance) associated with CKD could result in different metabolic routes for phenolic compounds from ASE. Phenolic compounds may undergo several distinct metabolic pathways and biotransformation steps by intestinal microbiota and host, including hydrolysis, conjugation by phase II enzymes, glucuronidation, demethylation and reduction reactions. Moreover, some of these steps may be altered by disrupted microbiota in CKD, impairing polyphenol biotransformation^56,57^. In contrast, CKD mice that received ASE showed greater excretion of 3.4-dihydroxyphenylacetic acid compared to the Control group. We emphasize that 3.4-dihydroxyphenylacetic acid is a metabolite from tyrosine and a precursor of p-CS, an important protein-bound uremic toxin. This observation suggests a change in the composition of the colonic microbiota in CKD, favoring the growth of proteolytic bacteria, particularly uremic toxin producing species^58^.

In both groups, hippuric acid (HA) was found to be the main metabolite in the urine; in fact, HA is one of the main phenolic acids excreted after the consumption of flavan-3-ols^59^. However, the concentration of this metabolite was two-fold higher in the plasma of CKD mice. It is noteworthy that this phenolic acid is considered as an uremic toxin because it can accumulate in the plasma of renal patients and promote toxic effects including neurological symptoms, metabolic acidosis, left ventricular hypertrophy, endothelial dysfunction, and glomerular sclerosis^60–62^.

As with the findings of our study, HA showed higher concentration in the plasma of uremic mice; on the other hand, urinary excretion of this metabolite was similar compared to the Control group^39^. The authors suggest that this phenomenon is related to dysbiosis and results in a higher load of glomerular filtrate, restoring the excretion rate to normal levels. Dysbiosis has often been associated with CKD and is characterized by qualitative and quantitative changes in the host microbiome profile, followed by changes in the protective function of the intestinal barrier. One of the most relevant changes in the CKD microbiome profile is a higher prevalence of proteolytic uremic solute producing bacteria and enzyme producing bacteria (such as urease and uricase). Dysbiosis is related to renal dysfunction, increased cardiovascular risk in CKD, uremic toxicity and inflammation^63–65^. Finally, we point out that changes in the intestinal microbiota may affect the metabolism of different components from diets, for example, phenolic compounds, and this fact may be responsible for individual variations in homeostasis and the effects exerted by different compounds, as reported in the present study.

This study, however, has some limitations. First, the adenine mouse model used in the present study mainly mimics tubulopathies that are not the most common causes of kidney failure in humans. Thus, further studies with other models of kidney failure, e.g., 5/6 subtotal nephrectomy or unilateral ureter obstruction (UUO) could be used to confirm the findings. The antihypertensive activity of ASE (as previously demonstrated in 2K-1C rats) could have contributed to nephroprotection, as found in the present study. Since blood pressure measurements were not performed, we cannot rule out this hypothesis. Adenine mice, however, do not consistently exhibit hypertension in contrast to mice with renal failure induced by 5/6 nephrectomy.

## Conclusion

Taking all results together, our data demonstrate that ASE could improve the treatment of renal failure through its antifibrotic and antioxidant activities. The reno-protective effect of ASE could be related to the inhibition of TGF- β1 pathway. CKD mice receiving ASE presented a distinct metabolite profile compared to the control mice receiving ASE. This result suggests different metabolization routes of phenolic compounds of ASE in vivo*.* Supplementation with Açai products might be an interesting nutritional strategy to improve the progression of kidney disease towards renal failure. Further studies are needed, however, to evidence this effect in patients with kidney disease.

## Supplementary Information

Supplementary Information.

## Data Availability

The data produced during the current study and the specific reagents (such as ASE) could be made available from the corresponding author upon reasonable request.

## Abbreviations

ASE: Açaí seed extract
BSA: Bovine serum albumin
BW: Body weight
CKD: Chronic kidney disease
Col-1α: Collagen 1α
CTGF: Connective tissue growth factor
CVD: Cardiovascular diseases
DNPH: 2,4-Dinitrophenylhydrazine
EIA: Enzyme-immunoassay
EMT: Epithelial to mesenchymal transition
FRAP: Ferric reducing ability of plasma
GAPDH: Glyceraldehyde-3-phosphate dehydrogenase
HA: Hippuric acid
HK2: Human kidney-2 cells
HPLC: High performance liquid chromatography
IS: Indoxyl sulfate
2K-1C: Two-kidneys one-clip
KIM-1: Kidney injury molecule-1
MDA: Malondialdehyde
NF-κB: Nuclear factor-kappa B
NGAL: Neutrophil gelatinase-associated lipocalin
NRF2: Erythroid 2-related factor 2
8-OH-dG: Hydroxydeoxyguanosine
p-CS: P-cresyl sulfate
PBUTs: Protein bound uremic toxins
ROS: Reactive oxygen species
α-SMA: α-Smooth muscle actin
SPE: Solid phase extraction
TBP: TATA-box binding protein
TGF-β1: Transforming growth factor β1
UV: Ultraviolet
