# Supplementary material for: Polyphenol-rich açaí seed extract exhibits reno-protective and anti-fibrotic activities in renal tubular cells and mice with kidney failure
Source: Sci Rep. 2022 Dec 2;12:20855. doi: 10.1038/s41598-022-24420-1 (PMC9718837; doi:10.1038/s41598-022-24420-1)
Supplement: Supplementary file 1 — Supplementary Information. [file 41598_2022_24420_MOESM1_ESM.pdf]

Supplementary Table 1.

Phenolic compounds in açaí seed extract (ASE) analyzed by HPLC-DAD-MALDI-TOF MS

**Compound**	Content (mg/100g)
Gallic acid	2.39 ± 0.16
3,4-Dihydroxybenzoic acid	3.41 ± 0.23
(+)-Catechin	419.30 ± 22.90
Syringic acid	76.18 ± 1.66
(-)-Epicatechin	473.96 ± 32.70
(-)-Epigallocatechin gallate	58.07 ± 1.26
Quercetin-3-*O*-rutinoside	10.83 ± 0.72
**Total**	1,044.14 ± 11.00

Values are expressed as mean ± standard deviation. Adapted from de Oliveira et al, 2015 and Soares et al, 2017.

References:

de Oliveira, P. R. B., da Costa, C. A., de Bem, G. F., Cordeiro, V. S. C., Santos, I. B., de Carvalho, L. C. R. M., da Conceição, E. P. S., Lisboa, P. C., Ognibene, D. T., Sousa, P. J. C., Martins, G. R., da Silva, A. J. R., de Moura, R. S., & Resende, A. C. (2015). Euterpe oleracea Mart.-Derived Polyphenols Protect Mice from Diet-Induced Obesity and Fatty Liver by Regulating Hepatic Lipogenesis and Cholesterol Excretion. *PloS One*, *10*(12), e0143721. https://doi.org/10.1371/journal.pone.0143721

Soares, E. R., Monteiro, E. B., de Bem, G. F., Inada, K. O. P., Torres, A. G., Perrone, D., Soulage, C. O., Monteiro, M. C., Resende, A. C., Moura-Nunes, N., Costa, C. A., & Daleprane, J. B. (2017). Up-regulation of Nrf2-antioxidant signaling by Açaí (Euterpe oleracea Mart.) extract prevents oxidative stress in human endothelial cells. Journal of Functional Foods, 37, 107–115. https://doi.org/10.1016/j.jff.2017.07.035

Supplementary Table 2.

Sequence of primers used for qPCR analysis

Gene name	Gene ID	Forward Primer	Reverse Primer
Primers used in animal experiments (Mus musculus)			
Tgfb1	21803	AGGGCTACCATGCCAACTTC	GTAACTGAGTTCTGACAGTG
Col1a1	12842	CAACATGGAGACAGGTCAGA	CTTGCAGTGATAGGTGATGT
Kim-1	171283	TCCACACATGTACCAACATCAA	GTCACAGTGCCATTCCAGTC
Tbp	21374	TGGTGTGCACAGGAGCCAAG	TTCACATCACAGCTCCCCAC
Primers used in cell experiments (Homo sapiens)			
Tgfb1	7040	AGGGCTACCATGCCAACTTC	GTAACTGAGTTCTGACAGTG
Col1a1	1277	CAACATGGAGACAGGTCAGA	CTTGCAGTGATAGGTGATGT
Acta2	59	GGACGCAGGTTCTCCAAACC	AAGTTCCACGGCTGCATTCC
Ctgf	1490	GTGCACCGC CAAAGATGG T	AAGGACTCTCCGCTGCGGTA
Gapdh	2597	CAACGGATTTGGTCGTATTGG	AACAATATCCACTTTACCAGA

Abbreviations: Acta2, α-smooth muscle actin, Col1a1: alpha-1 type 1 collagen, Ctgf, connective tissue growth factor, Gapdh, glyceraldehyde-3-Phosphate Dehydrogenase, Kim-1, kidney injury molecule-1, qPCR: Quantitative Polymerase Chain Reaction, Tbp: TATA-box binding protein, Tgfb1: Transforming Growth Factor beta 1.

Supplementary Table 3.

Gradient elution system used in phenolic compounds quantification by HPLC in plasma and urine of experimental mice

Time (min)	Eluent A (formic acid 0.3% and acetonitrile 1% in water) (%)	Eluent B (acetonitrile 1% in methanol) (%)
0	81.8	18.2
01	79.8	20.2
18	56.6	43.4
23	14.2	85.8
30	14.2	85.8
30.01	81.8	18.2
40.01	81.8	18.2

Supplementary Figure 1

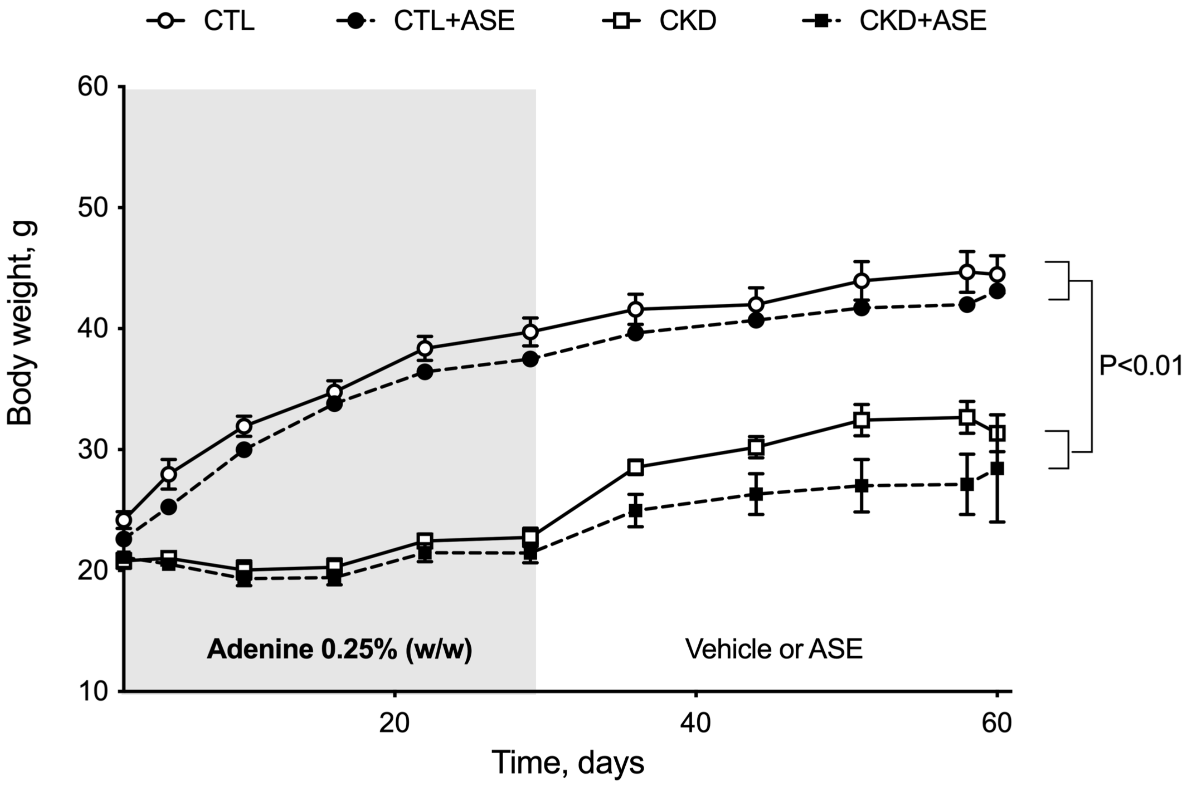

Supplementary Figure 1. Evolution of body weight of mice of the 4 experimental groups during the whole protocol.

Differences between groups were considered significant at the $P<0.05$ level.

Supplementary Figure 2

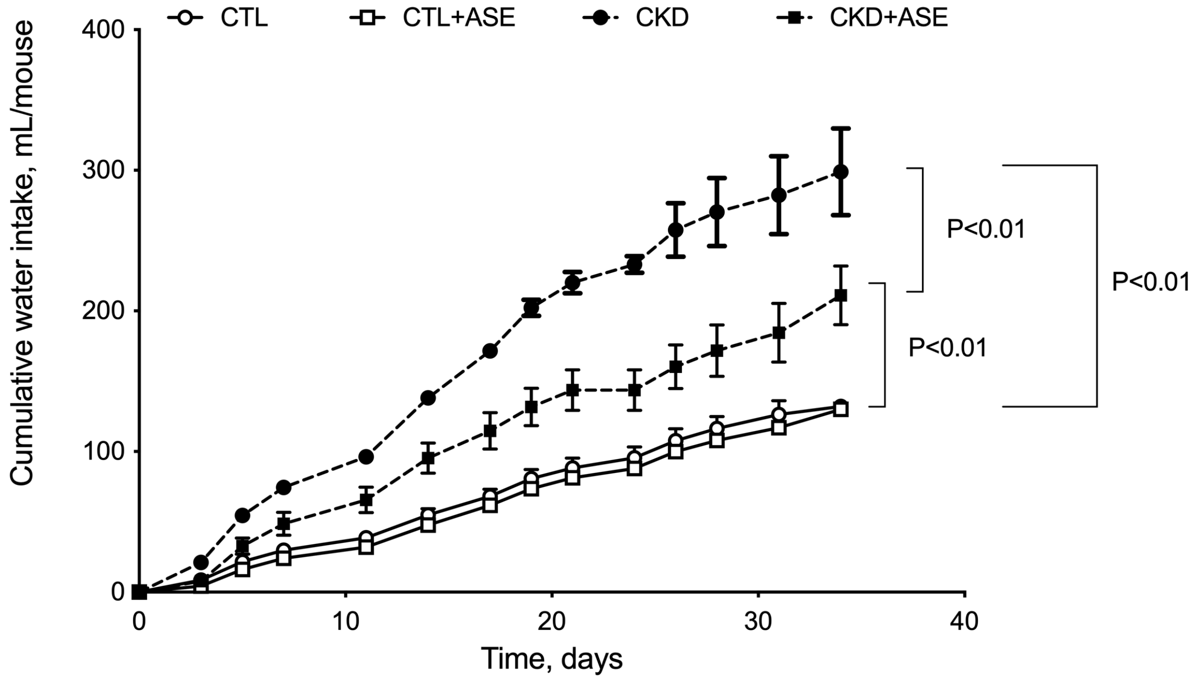

Supplementary Figure 2. **Cumulative water intake for the mice of the 4 experimental groups during the whole protocol.**

Note that water intake, as discussed in the manuscript, is a good proxy of the 24h diuresis of mice. Differences between groups were considered significant at the *P*<0.05 level.
